# An ortho­rhom­bic polymorph of 6-de­oxy-6-iodo-1,2:3,4-di-*O*-isopropyl­idene-α-d-galactopyran­oside

**DOI:** 10.1107/S1600536809029031

**Published:** 2009-07-25

**Authors:** Hoong-Kun Fun, Wei-Ching Liew, Sankappa Rai, Prakash Shetty, Arun M. Isloor

**Affiliations:** a X-ray Crystallography Unit, School of Physics, Universiti Sains Malaysia, 11800 USM, Penang, Malaysia; b Syngene International Ltd, Biocon Park, Plot Nos. 2 & 3, Bommasandra 4th Phase, Jigani Link Rd, Bangalore 560 100, India; c Department of Printing, Manipal Institute of Technology, Manipal 576 104, India; d Department of Chemistry, National Institute of Technology-Karnataka, Surathkal, Mangalore 575 025, India

## Abstract

The title compound, C_12_H_19_IO_5_, is the ortho­rhom­bic polymorph of a previously reported monoclinic form [Krajewski *et al.* (1987 ▶). *Bull. Pol. Acad. Sci. Chem.* **35**, 91–102]. The dihedral angles between the six-membered ring and the two five-membered rings are 67.66 (14) and 71.79 (13)°, whereas the dihedral angle between the five-membered rings is 74.41 (12)°, indicating that all three rings are twisted from each other. The six-membered ring has a twist-boat conformation while both of the five-membered rings have envelope conformations. The crystal structure is stabilized by a network of C—H⋯O contacts linking the mol­ecules into a two-dimensional array parallel to the *ab* plane.

## Related literature

For the monoclinic polymorph of the title compound, see: Krajewski *et al.* (1987 ▶). For the synthesis and biological evaluation of 6-substituted purines, see: Gambogi Braga *et al.* (2007 ▶). For halogenation reagent systems, see: Classon *et al.* (1988 ▶). For the synthesis of perosamine derivatives, see: Stevens *et al.* (1970 ▶). For the synthesis of labilose, see: Westwood *et al.* (1967 ▶). For ring conformations and ring puckering analysis, see: Boeyens (1978 ▶); Cremer & Pople (1975 ▶). For the stability of the temperature controller used in the data collection, see: Cosier & Glazer (1986 ▶).
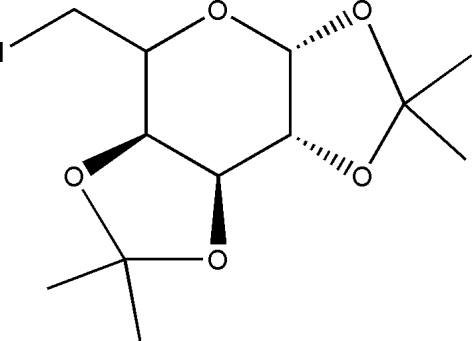


## Experimental

### 

#### Crystal data

C_12_H_19_IO_5_
*M* *_r_* = 370.17
Orthorhombic, 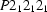

*a* = 7.3595 (1) Å
*b* = 11.5145 (2) Å
*c* = 16.9945 (2) Å
*V* = 1440.13 (4) Å^3^
*Z* = 4
Mo *K*α radiation
μ = 2.23 mm^−1^
*T* = 100 K
0.17 × 0.11 × 0.11 mm

#### Data collection

Bruker SMART APEXII CCD area-detector diffractometer
Absorption correction: multi-scan (**SADABS**; Bruker, 2005 ▶) *T*_min_ = 0.703, *T*_max_ = 0.785
27359 measured reflections
7509 independent reflections
6211 reflections with *I* > 2σ(*I*)
*R*_int_ = 0.046

#### Refinement

*R*[*F* ^2^ > 2σ(*F* ^2^)] = 0.035
*wR*(*F* ^2^) = 0.096
*S* = 1.07
7509 reflections
167 parameters
H-atom parameters constrained
Δρ_max_ = 0.87 e Å^−3^
Δρ_min_ = −1.24 e Å^−3^
Absolute structure: Flack (1983 ▶), 3286 Friedel pairs
Flack parameter: −0.020 (19)

### 

Data collection: *APEX2* (Bruker, 2005 ▶); cell refinement: *SAINT* (Bruker, 2005 ▶); data reduction: *SAINT*; program(s) used to solve structure: *SHELXTL* (Sheldrick, 2008 ▶); program(s) used to refine structure: *SHELXTL*; molecular graphics: *SHELXTL*; software used to prepare material for publication: *SHELXTL* and *PLATON* (Spek, 2009).

## Supplementary Material

Crystal structure: contains datablocks global, I. DOI: 10.1107/S1600536809029031/tk2509sup1.cif

Structure factors: contains datablocks I. DOI: 10.1107/S1600536809029031/tk2509Isup2.hkl

Additional supplementary materials:  crystallographic information; 3D view; checkCIF report

## Figures and Tables

**Table 1:** Hydrogen-bond geometry (Å, °)

*D*—H⋯*A*	*D*—H	H⋯*A*	*D*⋯*A*	*D*—H⋯*A*
C8—H8*B*⋯O2^i^	0.97	2.42	3.377 (3)	169
C12—H12*C*⋯O2^ii^	0.96	2.60	3.477 (4)	152

Symmetry codes: (i) 
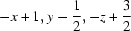
; (ii) 
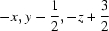
.

**Fig. 1.**
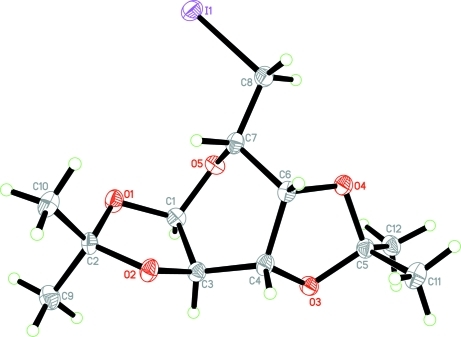
The molecular structure of (I), showing 50% probability displacement ellipsoids and the atom numbering scheme.

**Fig. 2.**
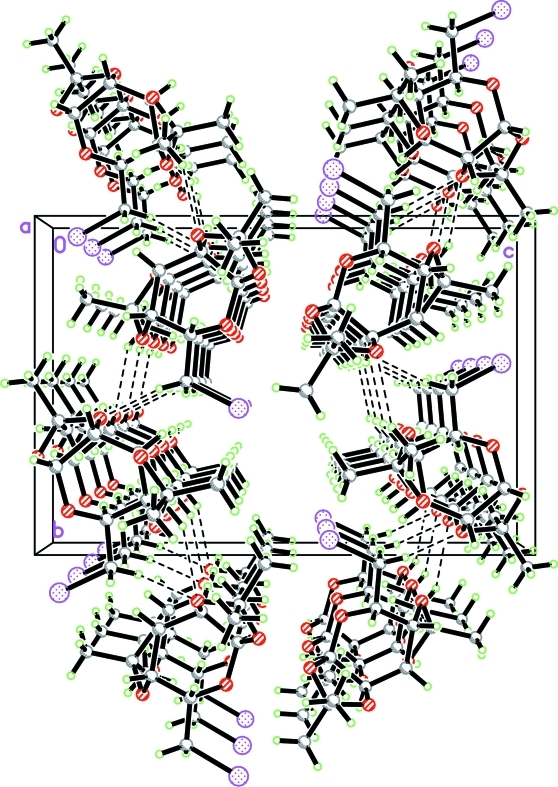
Crystal packing viewed along the a axis. The C-H···O contacts are shown as dashed lines.

**Fig. 3.**
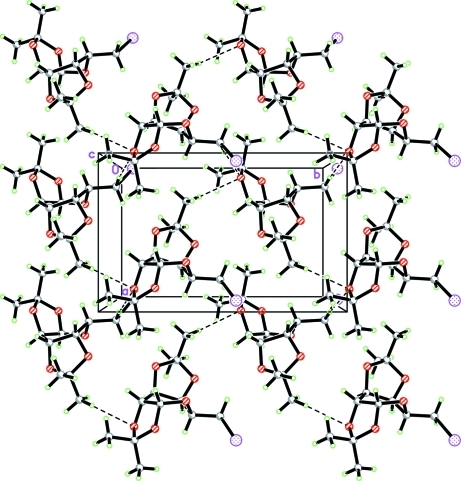
Crystal packing viewed along the c axis. The C-H···O contacts are shown as dashed lines.

## supplementary crystallographic information

### Comment

Libilomycin, an antibiotic which inhibits the growth of Gram-positive bacteria (Westwood *et al.*, 1967) and which is effective against certain tumor cells, is produced by the microorganism, streptomyces albosporeas (Stevens *et al.*, 1970), and contains 6-iodo,6-deoxy-1,2,3,4-di-*O*-isopropylidine-α-D-galactopyranoside as a part of its structure (Classon *et al.*, 1988). These intermediates are used for the synthesis of 6-substituted purines which show high activity against Leishmania amazonensis (Gambogi Braga *et al.*, 2007). These results prompted us to synthesize the title compound, (I).

Compound (I), Fig. 1, crystallized in the orthorhombic space group *P*2_1_2_1_2_1_ but has been reported previously (Krajewski *et al.*, 1987) in the monoclinic space group *P*2_1_, with a = 11.157 (2) Å, b = 20.047 (4) Å, c = 14.188 (2) Å and β = 107.67 (1)°. The dihedral angles between the six-membered ring systems, ring *A* (C1/C3/C4/C6/C7/O5), and the five-membered ring systems [rings *B* (C1—C3/O1—O2) and *C* (C4—C6/O3—O4)] are 67.66 (14)° and 71.79 (13)°, repectively. Moreover, the dihedral angle between rings *B* and *C* is 74.41 (12)° indicating that all the three rings are twisted from each other. Ring *A* adopts the twist-boat conformation (Boeyens, 1978; Cremer & Pople, 1975) with puckering amplitude Q = 0.629 (2) Å, φ = 75.3 (3)° and θ = 325.6 (2)°. On the other hand, each of rings *B* and *C* adopt an envelope conformation with flap atoms O2 and C5, respectively, but having different puckering parameters. For ring *B*, the puckering amplitude Q = 0.285 (2) Å and φ = 294.9 (5)° whereas for ring *C*, the puckering amplitude Q = 0.323 (3) Å and φ = 150.8 (4)°.

The crystal packing (Fig. 2 & Fig. 3) is consolidated by C8—H8B···O2 and C12—H12C···O2 contacts (Table 1) that link the molecules into a 2-D array parallel to the *ab* plane.

### Experimental

Triphenylphosphine (0.53 g, 1.9 mmol) and imidazole (0.4 g, 5.7 mmol) was added to the mixture of 1,2,3,4-di-*O*-isopropylidine-α-D-galactopyranoside (0.5 g, 1.9 mmol) in toluene: acetonitrile (2: 1, 10 ml). The mixture was heated to 70 °C. Iodine (0.6 g, 3.8 mmol) was then added portion-wise for a period of 30 min and mixture was further stirred for 2 hours. The completion of the reaction was confirmed by TLC (30% EtOAc/hexane, *R*_f_ - 0.6). The brown reaction mixture was concentrated under vacuum and the residue was purified by column chromatography using 25% ethylacetate in petroleum ether to get desired compound as white crystals. (Yield 600 mg, 83%, *m.p.* 334–336 K).

### Refinement

C-bound H atoms were positioned geometrically [C—H = 0.96–0.98 Å] and refined using a riding model, with *U*_iso_(H) = 1.2*U*_eq_(C) and 1.5*U*_eq_(methyl-C). A rotating group model was used for the methyl groups.

The maximum and minimum residual electron density peaks of 0.87 and -1.24 eÅ^-3^, respectively, were located 0.85 Å and 0.52 Å from the H8A and I1 atoms, respectively.

### Crystal data

**Table:**

C_12_H_19_IO_5_	*F*(000) = 736
*M**_r_* = 370.17	*D*_x_ = 1.707 Mg m^−^^3^
Orthorhombic, *P*2_1_2_1_2_1_	Mo *K*α radiation, λ = 0.71073 Å
Hall symbol: P 2ac 2ab	Cell parameters from 6517 reflections
*a* = 7.3595 (1) Å	θ = 3.0–30.1°
*b* = 11.5145 (2) Å	µ = 2.23 mm^−^^1^
*c* = 16.9945 (2) Å	*T* = 100 K
*V* = 1440.13 (4) Å^3^	Block, colourless
*Z* = 4	0.17 × 0.11 × 0.11 mm

### Data collection

**Table:**

Bruker SMART APEXII CCD area-detector diffractometer	7509 independent reflections
Radiation source: fine-focus sealed tube	6211 reflections with *I* > 2σ(*I*)
graphite	*R*_int_ = 0.046
φ and ω scans	θ_max_ = 37.5°, θ_min_ = 2.1°
Absorption correction: multi-scan (*SADABS*; Bruker, 2005)	*h* = −12→12
*T*_min_ = 0.703, *T*_max_ = 0.785	*k* = −19→17
27359 measured reflections	*l* = −28→29

### Refinement

**Table:**

Refinement on *F*^2^	Secondary atom site location: difference Fourier map
Least-squares matrix: full	Hydrogen site location: inferred from neighbouring sites
*R*[*F*^2^ > 2σ(*F*^2^)] = 0.035	H-atom parameters constrained
*wR*(*F*^2^) = 0.096	*w* = 1/[σ^2^(*F*_o_^2^) + (0.0418*P*)^2^ + 0.1348*P*] where *P* = (*F*_o_^2^ + 2*F*_c_^2^)/3
*S* = 1.07	(Δ/σ)_max_ = 0.003
7509 reflections	Δρ_max_ = 0.87 e Å^−^^3^
167 parameters	Δρ_min_ = −1.24 e Å^−^^3^
0 restraints	Absolute structure: Flack (1983), 3286 Friedel pairs
Primary atom site location: structure-invariant direct methods	Flack parameter: −0.020 (19)

### Special details

**Table:**

Experimental. The crystal was placed in the cold stream of an Oxford Cyrosystems Cobra open-flow nitrogen cryostat (Cosier & Glazer, 1986) operating at 100.0 (1) K.
Geometry. All e.s.d.'s (except the e.s.d. in the dihedral angle between two l.s. planes) are estimated using the full covariance matrix. The cell e.s.d.'s are taken into account individually in the estimation of e.s.d.'s in distances, angles and torsion angles; correlations between e.s.d.'s in cell parameters are only used when they are defined by crystal symmetry. An approximate (isotropic) treatment of cell e.s.d.'s is used for estimating e.s.d.'s involving l.s. planes.
Refinement. Refinement of *F*^2^ against ALL reflections. The weighted *R*-factor *wR* and goodness of fit *S* are based on *F*^2^, conventional *R*-factors *R* are based on *F*, with *F* set to zero for negative *F*^2^. The threshold expression of *F*^2^ > σ(*F*^2^) is used only for calculating *R*-factors(gt) *etc*. and is not relevant to the choice of reflections for refinement. *R*-factors based on *F*^2^ are statistically about twice as large as those based on *F*, and *R*- factors based on ALL data will be even larger.

### Fractional atomic coordinates and isotropic or equivalent isotropic displacement parameters (Å^2^)

**Table:**

	*x*	*y*	*z*	*U*_iso_*/*U*_eq_	
I1	0.48767 (2)	−0.062287 (16)	0.585388 (10)	0.02322 (5)	
O1	0.3941 (3)	0.30650 (19)	0.54871 (11)	0.0202 (4)	
O2	0.4126 (2)	0.41053 (17)	0.66190 (11)	0.0174 (4)	
O3	−0.0095 (3)	0.31453 (16)	0.74636 (10)	0.0169 (3)	
O4	0.0869 (3)	0.13209 (18)	0.77122 (12)	0.0181 (4)	
O5	0.2158 (2)	0.16776 (16)	0.61095 (11)	0.0162 (3)	
C1	0.2269 (3)	0.2856 (2)	0.58845 (16)	0.0166 (4)	
H1A	0.1256	0.3041	0.5532	0.020*	
C2	0.4873 (3)	0.4031 (2)	0.58404 (14)	0.0167 (4)	
C3	0.2281 (3)	0.3722 (2)	0.65788 (15)	0.0166 (4)	
H3A	0.1476	0.4379	0.6466	0.020*	
C4	0.1835 (3)	0.3209 (3)	0.73797 (15)	0.0167 (4)	
H4A	0.2335	0.3710	0.7793	0.020*	
C5	−0.0464 (3)	0.2160 (2)	0.79401 (15)	0.0165 (4)	
C6	0.2485 (3)	0.1941 (2)	0.75132 (13)	0.0152 (5)	
H6A	0.3359	0.1907	0.7948	0.018*	
C7	0.3309 (3)	0.1412 (2)	0.67660 (15)	0.0165 (5)	
H7A	0.4522	0.1737	0.6677	0.020*	
C8	0.3433 (4)	0.0106 (3)	0.68407 (16)	0.0194 (5)	
H8A	0.2220	−0.0221	0.6864	0.023*	
H8B	0.4059	−0.0092	0.7325	0.023*	
C9	0.4481 (4)	0.5139 (3)	0.53734 (17)	0.0235 (6)	
H9A	0.3193	0.5267	0.5353	0.035*	
H9B	0.4945	0.5057	0.4848	0.035*	
H9C	0.5058	0.5787	0.5626	0.035*	
C10	0.6874 (4)	0.3785 (3)	0.59115 (18)	0.0220 (5)	
H10A	0.7049	0.3032	0.6142	0.033*	
H10B	0.7430	0.4364	0.6240	0.033*	
H10C	0.7421	0.3804	0.5399	0.033*	
C11	−0.0254 (4)	0.2444 (3)	0.88146 (15)	0.0241 (5)	
H11A	0.0946	0.2739	0.8910	0.036*	
H11B	−0.0440	0.1753	0.9120	0.036*	
H11C	−0.1136	0.3018	0.8963	0.036*	
C12	−0.2321 (4)	0.1723 (3)	0.77260 (18)	0.0205 (5)	
H12A	−0.2307	0.1435	0.7196	0.031*	
H12B	−0.3183	0.2346	0.7766	0.031*	
H12C	−0.2662	0.1109	0.8078	0.031*	

### Atomic displacement parameters (Å^2^)

**Table:**

	*U*^11^	*U*^22^	*U*^33^	*U*^12^	*U*^13^	*U*^23^
I1	0.02406 (8)	0.02602 (9)	0.01959 (7)	0.00713 (7)	0.00446 (7)	0.00100 (6)
O1	0.0212 (9)	0.0223 (10)	0.0170 (8)	−0.0053 (8)	0.0033 (7)	−0.0029 (8)
O2	0.0136 (7)	0.0220 (10)	0.0167 (8)	−0.0033 (6)	0.0016 (6)	−0.0022 (7)
O3	0.0124 (7)	0.0161 (7)	0.0222 (7)	−0.0003 (6)	0.0014 (6)	0.0023 (6)
O4	0.0133 (8)	0.0188 (9)	0.0223 (9)	0.0002 (6)	0.0036 (7)	0.0023 (7)
O5	0.0169 (8)	0.0149 (9)	0.0166 (7)	−0.0011 (6)	−0.0038 (6)	0.0004 (7)
C1	0.0177 (9)	0.0178 (12)	0.0143 (9)	−0.0010 (8)	−0.0028 (9)	−0.0001 (9)
C2	0.0173 (9)	0.0193 (10)	0.0134 (8)	−0.0026 (8)	0.0025 (11)	0.0023 (8)
C3	0.0145 (10)	0.0180 (12)	0.0172 (10)	−0.0018 (8)	0.0006 (8)	−0.0008 (9)
C4	0.0127 (9)	0.0207 (13)	0.0167 (10)	−0.0020 (8)	0.0012 (8)	−0.0009 (9)
C5	0.0150 (10)	0.0179 (12)	0.0166 (10)	0.0003 (8)	0.0014 (8)	0.0017 (8)
C6	0.0125 (9)	0.0197 (13)	0.0135 (10)	−0.0002 (8)	−0.0011 (7)	0.0007 (9)
C7	0.0127 (10)	0.0218 (13)	0.0149 (10)	−0.0004 (8)	−0.0003 (8)	0.0005 (9)
C8	0.0186 (11)	0.0227 (14)	0.0170 (11)	0.0044 (9)	0.0011 (9)	0.0041 (10)
C9	0.0251 (13)	0.0236 (14)	0.0218 (12)	−0.0012 (10)	0.0004 (10)	0.0054 (11)
C10	0.0175 (10)	0.0292 (15)	0.0193 (11)	0.0012 (9)	0.0031 (10)	0.0007 (11)
C11	0.0227 (13)	0.0313 (15)	0.0182 (10)	0.0003 (11)	0.0021 (10)	−0.0010 (10)
C12	0.0170 (11)	0.0200 (14)	0.0245 (13)	−0.0021 (9)	0.0014 (9)	0.0036 (11)

### Geometric parameters (Å, °)

**Table:**

I1—C8	2.155 (3)	C5—C11	1.530 (4)
O1—C1	1.424 (3)	C6—C7	1.533 (4)
O1—C2	1.438 (3)	C6—H6A	0.9800
O2—C3	1.430 (3)	C7—C8	1.512 (4)
O2—C2	1.435 (3)	C7—H7A	0.9800
O3—C5	1.420 (3)	C8—H8A	0.9700
O3—C4	1.429 (3)	C8—H8B	0.9700
O4—C6	1.428 (3)	C9—H9A	0.9600
O4—C5	1.431 (3)	C9—H9B	0.9600
O5—C1	1.412 (3)	C9—H9C	0.9600
O5—C7	1.434 (3)	C10—H10A	0.9600
C1—C3	1.545 (4)	C10—H10B	0.9600
C1—H1A	0.9800	C10—H10C	0.9600
C2—C10	1.505 (4)	C11—H11A	0.9600
C2—C9	1.530 (4)	C11—H11B	0.9600
C3—C4	1.519 (4)	C11—H11C	0.9600
C3—H3A	0.9800	C12—H12A	0.9600
C4—C6	1.554 (4)	C12—H12B	0.9600
C4—H4A	0.9800	C12—H12C	0.9600
C5—C12	1.501 (4)		
			
C1—O1—C2	110.17 (19)	C7—C6—H6A	110.4
C3—O2—C2	107.52 (19)	C4—C6—H6A	110.4
C5—O3—C4	106.8 (2)	O5—C7—C8	108.2 (2)
C6—O4—C5	107.3 (2)	O5—C7—C6	109.1 (2)
C1—O5—C7	112.42 (19)	C8—C7—C6	110.4 (2)
O5—C1—O1	109.9 (2)	O5—C7—H7A	109.7
O5—C1—C3	114.4 (2)	C8—C7—H7A	109.7
O1—C1—C3	104.4 (2)	C6—C7—H7A	109.7
O5—C1—H1A	109.3	C7—C8—I1	110.61 (18)
O1—C1—H1A	109.3	C7—C8—H8A	109.5
C3—C1—H1A	109.3	I1—C8—H8A	109.5
O2—C2—O1	104.41 (18)	C7—C8—H8B	109.5
O2—C2—C10	108.2 (2)	I1—C8—H8B	109.5
O1—C2—C10	110.8 (2)	H8A—C8—H8B	108.1
O2—C2—C9	110.8 (2)	C2—C9—H9A	109.5
O1—C2—C9	109.8 (2)	C2—C9—H9B	109.5
C10—C2—C9	112.5 (2)	H9A—C9—H9B	109.5
O2—C3—C4	106.4 (2)	C2—C9—H9C	109.5
O2—C3—C1	104.0 (2)	H9A—C9—H9C	109.5
C4—C3—C1	115.6 (2)	H9B—C9—H9C	109.5
O2—C3—H3A	110.2	C2—C10—H10A	109.5
C4—C3—H3A	110.2	C2—C10—H10B	109.5
C1—C3—H3A	110.2	H10A—C10—H10B	109.5
O3—C4—C3	108.9 (2)	C2—C10—H10C	109.5
O3—C4—C6	104.1 (2)	H10A—C10—H10C	109.5
C3—C4—C6	115.4 (2)	H10B—C10—H10C	109.5
O3—C4—H4A	109.4	C5—C11—H11A	109.5
C3—C4—H4A	109.4	C5—C11—H11B	109.5
C6—C4—H4A	109.4	H11A—C11—H11B	109.5
O3—C5—O4	104.70 (19)	C5—C11—H11C	109.5
O3—C5—C12	107.7 (2)	H11A—C11—H11C	109.5
O4—C5—C12	109.4 (2)	H11B—C11—H11C	109.5
O3—C5—C11	111.4 (2)	C5—C12—H12A	109.5
O4—C5—C11	109.8 (2)	C5—C12—H12B	109.5
C12—C5—C11	113.5 (2)	H12A—C12—H12B	109.5
O4—C6—C7	109.1 (2)	C5—C12—H12C	109.5
O4—C6—C4	104.4 (2)	H12A—C12—H12C	109.5
C7—C6—C4	112.0 (2)	H12B—C12—H12C	109.5
O4—C6—H6A	110.4		

### Hydrogen-bond geometry (Å, °)

**Table:**

*D*—H···*A*	*D*—H	H···*A*	*D*···*A*	*D*—H···*A*
C8—H8B···O2^i^	0.97	2.42	3.377 (3)	169
C12—H12C···O2^ii^	0.96	2.60	3.477 (4)	152

Symmetry codes: (i) −*x*+1, *y*−1/2, −*z*+3/2; (ii) −*x*, *y*−1/2, −*z*+3/2.

## References

[bb1] Boeyens, J. C. A. (1978). *J. Cryst. Mol. Struct.* **8**, 317–320.

[bb2] Bruker (2005). *APEX2*, *SAINT* and *SADABS* Bruker AXS Inc., Madison, Wisconsin, USA.

[bb3] Classon, B., Liu, Z. & Samuelsson, B. (1988). *J. Org. Chem.* **53**, 6126–6130.

[bb4] Cosier, J. & Glazer, A. M. (1986). *J. Appl. Cryst.* **19**, 105–107.

[bb5] Cremer, D. & Pople, J. A. (1975). *J. Am. Chem. Soc.* **97**, 1354–1358.

[bb6] Flack, H. D. (1983). *Acta Cryst.* A**39**, 876–881.

[bb7] Gambogi Braga, F., Soares Coimbra, E., De Oliveira Matos, M., Lino Carmo, A. M., Damato Cancio, M. & Da Silva, A. D. (2007). *Eur. J. Med. Chem.* **42**, 530–537. 10.1016/j.ejmech.2006.10.014 17156894

[bb8] Krajewski, J. W., Gluzinski, P., Jarosz, S., Bleidelis, J., Mishnyov, A. & Kemme, A. (1987). *Bull. Pol. Acad. Sci. Chem.* **35**, 91–102.

[bb9] Sheldrick, G. M. (2008). *Acta Cryst.* A**64**, 112–122. 10.1107/S0108767307043930 18156677

[bb10] Spek, A. L. (2009). *Acta Cryst* D**65**, 148–155. 10.1107/S090744490804362X PMC2631630 19171970

[bb11] Stevens, C. L., Glinski, P. R., Taylor, K. G., Blumberg, P. & Gupta, S. K. (1970). *J. Am. Chem. Soc* **92**, 3160–3168. 10.1021/ja00713a039 5446955

[bb12] Westwood, J. H., Chalk, R. C., Ball, D. H. & Long, L. (1967). *J. Org. Chem.* **32**, 1643–1644. 10.1021/jo01280a089 6041437

